# Unifloral Autumn Heather Honey from Indigenous Greek *Erica manipuliflora* Salisb.: SPME/GC-MS Characterization of the Volatile Fraction and Optimization of the Isolation Parameters

**DOI:** 10.3390/foods10102487

**Published:** 2021-10-17

**Authors:** Marinos Xagoraris, Foteini Chrysoulaki, Panagiota-Kyriaki Revelou, Eleftherios Alissandrakis, Petros A. Tarantilis, Christos S. Pappas

**Affiliations:** 1 Laboratory of Chemistry, Department of Food Science and Human Nutrition, Agricultural University of Athens, 75 Iera Odos, 11855 Athens, Greece; mxagor@aua.gr (M.X.); fay_chrysoulaki@aua.gr (F.C.); p.revelou@aua.gr (P.-K.R.); ptara@aua.gr (P.A.T.); 2 Laboratory of Quality and Safety of Agricultural Products, Landscape and Environment, Department of Agriculture, Hellenic Mediterranean University, Stavromenos, 71410 Heraklion, Greece; ealiss@hmu.gr; 3 Institute of Agri-Food and Life Sciences Agro-Health, Hellenic Mediterranean University Research Center, Stavromenos, 71410 Heraklion, Greece

**Keywords:** autumn heather honey, *Erica manipuliflora* Salisb., volatiles, gas chromatography-mass spectrometry, solid-phase microextraction, optimization, response surface methodology

## Abstract

For long heather honey has been a special variety due to its unique organoleptic characteristics. This study aimed to characterize and optimize the isolation of the dominant volatile fraction of Greek autumn heather honey using solid-phase microextraction (SPME) followed by gas chromatography-mass spectrometry (GC-MS). The described approach pointed out 13 main volatile components more closely related to honey botanical origin, in terms of occurrence and relative abundance. These volatiles include phenolic compounds and norisoprenoids, with benzaldehyde, safranal and *p*-anisaldehyde present in higher amounts, while ethyl 4-methoxybenzoate is reported for the first time in honey. Then, an experimental design was developed based on five numeric factors and one categorical factor and evaluated the optimum conditions (temperature: 60 °C, equilibration time: 30 min extraction time: 15 min magnetic stirrer velocity: 100 rpm sample volume: 6 mL water: honey ratio: 1:3 (*v*/*w*)). Additionally, a validation test set reinforces the above methodology investigation. Honey is very complex and variable with respect to its volatile components given the high diversity of the floral source. As a result, customizing the isolation parameters for each honey is a good approach for streamlining the isolation volatile compounds. This study could provide a good basis for future recognition of monofloral autumn heather honey.

## 1. Introduction

Honey bees (*Apis mellifera* L.) are primary pollinators with an important role in ecosystem conservation [1], offering many services and products, such as honey. Honey through the centuries has always been a vital food for humans, with many health properties [2,3]. The Mediterranean region, specifically Greece pronounces a set of several common and rare monofloral honeys in international markets [4]. Additionally, nowadays few rare honeys, like heather, have become increasingly well-known for their special characteristics and have received several awards in national and international food quality or taste competitions [5]. The term “heather” is used for plant species belonging to *Erica* and *Calluna* genera. However, this term is used to describe the honey produced from *Calluna vulgaris* (L.) Hull and not from other Ericaceae botanical sources [6]. In relation to honey from common species, including *Erica arborea* L., *Erica carnea* L., and *Erica cinerea* L., the given names are “Tree heath”, “Spring heather”, and “Bell heather”, respectively [6].

Greek flora includes four Ericaceae nectar-secretion bee plants. Two of them are spring flowering species including *Erica arborea* L., and *Rhododendron* sp. while the other two (*Erica manipuliflora* Salisb. and *Arbutus unedo* L.) bloom in autumn. *Erica manipuliflora* is indigenous in Greece and is known as “autumn heather”, while the traditional term used is “sousoura”. However, honey from *E. manipuliflora* should not be confused with other heather honeys produced during autumn, including from *C. vulgaris*, and *Erica multiflora* L. Monofloral autumn heather honey can be quite easily produced [7], as its collection period does not coincide with the blooming of other bee plants, with the exception of *A. unedo* honey, which blooms in late autumn and its blooming period follows that of *E. manipuliflora*.

Greek autumn heather honey is well-known for its extraordinary aroma profile, characterized by perfume reminiscent “caramel” notes, which is worth studying since data for this honey variety are scarce. In the last twenty years, just two studies [8,9] have dealt with the volatile fraction of *E. manipuliflora* honey. However, there are numerous studies concerning heather honey [7,10,11,12,13,14,15,16,17]. As shown in a review study [18], the above studies refer to different botanical species, geographical origin, number of samples, isolation, and analysis procedures. 

The volatile isolation method is usually followed by gas chromatography-mass spectrometry (GC-MS), and plays a significant role in the qualitative and quantitative determination of volatiles. Solid-phase microextraction (SPME) as a volatile fraction extraction methodology constitutes a simple procedure with no pre-treatment of samples and environmentally friendly solvents [19]. The main factors, including temperature, equilibration time, extraction time, sample volume, water-honey ratio, and magnetic stirring velocities contribute simultaneously to the isolation of volatiles, sometimes synergistically [20]. For this reason, it is necessary to study all-factors-at-a-time, in terms of their effectiveness of volatiles isolation. This may be possible by using multivariate statistic techniques, like response surface methodology (RSM) [20,21].

The aim of the present study was the identification and semi-quantification of the volatile fraction of indigenous monofloral Greek autumn heather honey from *E. manipuliflora*. The main SPME factors were simultaneously examined for their potential to isolate the dominant volatile fraction and each molecule separately using RSM.

## 2. Materials and Methods

### 2.1. Honey Samples

The analyses of volatiles were carried out to 25 honey samples provided directly by Greek beekeepers. Samples were produced during the 2019–2021 harvest period. The botanical origin was assessed by the beekeepers and then confirmed by melissopalynological [22], and physiochemical analysis [23,24], as previously described [25]. Floral origin was confirmed firstly according to European [26] and secondly according to the more strict Greek [27] legislation (sum of fructose and glycose not less than 60% *w*/*w*; sucrose content not more than 5% *w*/*w*; moisture content not more than 20% *w*/*w*; electrical conductivity not more than 800 (μS cm^−1^); diastase activity (Schade scale) not less than 8; HMF not more than 40 mg kg^−1^; heather pollen not less than 45%). Honey samples were kept in the dark at 4 °C in hermetically closed glass bottles until further analysis.

### 2.2. Experimental Design

A central composite design (CCD) was used combined with RSM methodology by Box and Wilson [28]. A flexible design structure was constructed to accommodate a custom model, with numeric and categorical independent factors and irregular constrained regions. Five numeric factors (A, B, C, D, and E) and one categorical factor (F) were analyzed by a quadratic design domain. A total of 38 runs were determined by a selection criterion chosen during the experimental design (Table 1).

Τhe responses of the volatile compounds expressed as chromatographic area (%) were used as dependent variables. For this purpose, a randomly selected sample was used for response prediction. The model’s fitness was confirmed by analysis of variance (ANOVA) and the determination coefficient (R^2^) using *p*-values. Dependent variables were also confirmed by the Box-Cox, correlations, and normality of residuals statistical tests. All possible optimized solutions, for (a) volatile profile and (b) each volatile molecule separately were evaluated by maximizing desirability indices. The robustness of the model was validated with response data of 24 samples according to the optimum SPME solution. 

Statistical analysis was carried out using Desing-Expert 11.0.5.0 (Stat-Ease, Inc., Minneapolis, MN, USA).

### 2.3. Isolation and Analysis of Volatile Compounds

Isolation of the volatile fraction was done based on experimental design layout run (Table 1) using a manual holder with triple-phase divinylbenzene/carboxen/polydimethylsiloxane (DVB/CAR/PDMS) fiber 50/30 μm (Supelco, Bellefonte, PA, USA) with length of 1 cm. Before each analysis, fibers were conditioned at 270 °C. Moreover, a blank sample was performed for cleaning from previous volatile residues. Then, a predetermined volume ratio of water: Honey (*v*/*w*) was transferred in 15 mL screw top (22.7 × 86 mm) vials with PTFE/silicone septa and a portion of 20 μL (300 μg mL^−1^ in methanol) of benzophenone (Alfa Aesar, Kandal, Germany) was added as an internal standard.

RSM experiments were performed using a Trace Ultra gas chromatograph (GC) (Thermo Scientific Inc., Waltham, MA, USA), coupled with a mass spectrometer (MS) (DSQII, Thermo Scientific Inc., Waltham, MA, USA). GC-MS was performed with a Restek Rtx-5MS (30 m × 0.25 mm i.d., 0.25 μm film thickness) chromatography column with helium as carrier gas at a 1 mL min^−1^ rate. The chromatography conditions and temperature program have been previously described [29]. In brief, the GC inlet temperature 260 °C in the splitless mode for 3 min, with a 0.8 mm injector liner (SGE International Pty Ltd., Ringwood, Australia). Oven temperature was adapted to 40 °C for 6 min, then increased to 120 °C at a rate of 5 °C min^−1^, followed by an increment of 3 °C min^−1^ up to 160 °C and up to 250 °C with a step of 15 °C min^−1^. Finally, the temperature of 250 °C was kept constant for 1 min. The transfer line and injector temperatures were maintained at 290 and 220 °C, respectively. Electron impact was 70 eV, and mass spectra were recorded at the 35–650 mass range.

The peak identification was achieved with the Wiley 275 mass spectra library, and the arithmetic index provided by Adams [30]. Retention Index (RI) values of volatile compounds were calculated using n-alkane (C8–C20) standards (Supelco, Bellefonte, PA, USA). The isolated compounds were semi-quantified against the internal standard (benzophenone) and expressed as mg kg^−1^ of honey. All samples were analyzed in triplicate.

## 3. Results and Discussion

### 3.1. Evaluation of Isolated Volatile Compounds

In total, 49 volatile compounds were identified, including esters, hydrocarbons, alcohols, aldehydes, ketones, acids, terpenoids, and others (Table 2).

Esters have been encountered almost always in all blossom and honeydew honeys with some of them being dominant volatiles [18]. In our results, most esters were detected in small amounts except for methyl nonanoate. However, methyl nonanoate has been reported at much higher concentrations in honeydew honey, like fir and pine [29]. This presence could occur in the collection period of pine honey by the bees in October. Moreover, methyl octanoate and methyl dodecanoate can be related to the above conjecture [29]. Ethyl 4-methoxybenzoate was a derivative coming from *p*-anisic acid which has been reported in *Erica arborea* L. honey [10] by a Likens-Nickerson steam distillation (L-N) isolation technique. However, ethyl 4-methoxybenzoate was worth studying as it has not been detected in other botanical sources yet. 

Hydrocarbons were detected in most samples with undecane having the highest average compared to the rest. This class of volatiles is very common among honeys [18].

The chemical group of alcohols including 5-(3,3-dimethyloxiran-2-yl)-3-methylpent-1-en-3-ol (syn: *cis*-Linalool oxide) [11,17] and 2-phenylethan-1-ol [10,13,19] have been previously identified as dominant volatiles compounds of citrus, acacia, chestnut, and thyme honeys [18]. The compound, 3,4,5-trimethylphenol, has been previously described as one of the major volatile compounds of heather honey from Poland [14]. Furthermore, 4,6,10,10-tetramethyl-5-oxatricyclo[4.4.0.01,4]dec-2-en-7-ol and 6,6-dimethyl-5-methylenebicyclo[2.2.1]heptan-2-ol (6-camphenol) have been reported in *Erica* spp. honeys from Iberian Peninsula [17]. Nevertheless, the latter was not identified in all of our samples.

Aldehydes were detected in all samples, in smaller or larger amounts. Octanal, nonanal, and decanal were present in small quantities and are considered as important components of honeydew honey volatile profile [18]. Benzaldehyde [11,19] and 2-phenylacetaldehyde [13,16,17] were detected in all samples at a remarkable concentration. In addition, 2,6,6-trimethylcyclohexa-1,3-diene-1-carbaldehyde (safranal) [7]; 4-methoxybenzaldehyde (p-anisaldehyde) [10], and furan-2-carbaldehyde (furfural) [13] have been attributed to heather honey. 

Ketones include many degraded carotenoids related to heather honey. Some of these compounds, such as 3,5,5-trimethylcyclohex-2-en-1-one (a-isophorone); 2,6,6-trimethylcyclohex-2-ene-1,4-dione (4-oxoisophorone); 2-hydroxy-3,5,5-trimethylcyclohex-2-en-1-one (2-hydroxyisophorone); (E)-1-(2,6,6-trimethylcyclohexa-1,3-dien-1-yl)but-2-en-1-one (β-damascenone); (E)-1,6,6-trimethyl-7-(3-oxobut-1-en-1-yl)-3,8-dioxatricyclo[5.1.0.02,4]octan-5-one; and (E)-4-(2,6,6-trimethylcyclohexa-1,3-dien-1-yl)but-3-en-2-one [10,13,14,31] are known heather honey compounds, all of which have been detected in our samples. Notably, 1-(furan-2-yl)ethan-1-one was found in all samples.

Terpenoids, acids, and other compounds do not include significant volatile compounds of heather honey, except for 1,1,5-trimethyl-1,2-dihydronaphthalene and 8-isopropyl-1-methyl-1,2,3,4-tetrahydronaphthalene, that have been identified in another study as well [17].

Other studies refer to hotrienol, *cis*-linalool oxide, and 2-phenylacetaldehyde as the main volatile compounds of *Erica* spp. honey [13,17,19]. However, this is not confirmed by our samples. Hotrienol was not detected in any of our samples. Oxide of cis-linalool was linked with hive atmospheres or combustion of wood/vegetation during beekeeping activity [32], and 2-phenylacetaldehyde also had been reported in relevant concentrations, while some studies attribute this molecule to long-term storage by enzymatic catalysis of phenylalanine or heat treatment [33]. Furan derivatives identified in some of our samples, emanate from thermal processing and/or prolonged storage [34,35,36] and cannot be related to honey botanical origin.

### 3.2. Optimization of Each Dominant Volatile Compound

Several SPME conditions (A: Temperature; B: Equilibration time; C: Extraction time; D: Magnetic stirrer velocity; E: Sample volume; F: water: honey ratio) were investigated to determine the most suitable conditions for each volatile compound. A total of 13 volatile compounds were chosen for optimization (responses R1-R13) (Table 3). These compounds were selected as they constitute dominant and characteristic responses of autumn heather honey.

Prior to undertaking the processing steps, data for each volatile compound were confirmed by normal distribution, Box-Cox test, determination of coefficient (R^2^) and ANOVA (Table 4). The condition number of coefficient matrix (<10) did nοt indicate multicollinearity. Additionally, all responses followed the normal distribution. Box-Cox test provides a guideline for selecting the correct power law transformation. If the 95% confidence interval around this lambda includes 1.00, it does not require a specific transformation. Table 4 shows the lambda values at the 95% confidence range, as well as the current lambda. R-square (R^2^) constitutes a measure of the amount of variation around the mean explained by the model. The ANOVA in this case confirms the adequacy of the model (*p*-value < 0.05) and indicated whether the model terms were significant. Significant model terms may have a real effect on the response. 

ANOVA results showed many considerable independent SPME conditions, while some of them could contribute in combination. At the same time, equations were developed in terms of coded factors that can be used to make predictions about the response for given levels of each factor. By default, the high levels of the factors are coded as +1 and the low levels are coded as −1. The coded equation is useful for identifying the relative impact of the factors by comparing the factor coefficients. However, these equations should be considered with caution because it is not safe to use them as panacea for modeling future responses. In this case, all these results are presented in Table 5. 

After these steps, optimization models were developed based on each volatile molecule. The results are presented in Table 6 and were evaluated by desirability indices. A high level of ideal cases is coded as 1 and low level as zero. A predicted mean for each volatile response is also included. 

Experimental findings showed that optimum conditions of some volatiles required the maximum value of model terms. However, this conclusion is overturned by extraction time and magnetic stirrer velocity. As previously described, extraction time was a significant parameter, along with magnetic stirrer velocity, which in some cases, allowed better isolation of some compounds [20], whilst usually shortened the equilibration time. 

### 3.3. Optimization and Validation of Dominant Volatile Compounds

The optimum conditions proposed for dominant volatile compounds of autumn heather honey were A: 60 °C B: 30 min C: 15 min D: 100 rpm E: 6 mL F: 1:3 (*v*/*w*). Predicted mean (% Area) was estimated for benzaldehyde (4.53%), 3,5,5-trimethylcyclohex-2-en-1-one (0.88%), 2,6,6-trimethylcyclohex-2-ene-1,4-dione (1.64%), 2-hydroxy-3,5,5-trimethylcyclohex-2-en-1-one (0.80%), 2,6,6-trimethylcyclohexa-1,3-diene-1-carbaldehyde (1.29%), 4-methoxybenzaldehyde (12.21%), 3,4,5-trimethylphenol (0.12%), 1,1,5-trimethyl-1,2-dihydronaphthalene (0.66%), (E)-1-(2,6,6-trimethylcyclohexa-1,3-dien-1-yl)but-2-en-1-one (0.15%), (E)-1,6,6-trimethyl-7-(3-oxobut-1-en-1-yl)-3,8-dioxatricyclo[5.1.0.02,4]octan-5-one (0.71%), ethyl 4-methoxybenzoate (2.88%), (E)-4-(2,6,6-trimethylcyclohexa-1,3-dien-1-yl)but-3-en-2-one (0.77%), and 4,6,10,10-tetramethyl-5-oxatricyclo[4.4.0.01,4]dec-2-en-7-ol (0.97%). Moreover, the desirability of optimized model was calculated at 1.000.

The validation of the above results was carried out with a test set of 24 samples. All responses (R1-R13) were isolated in all samples with the confirmed optimum conditions of the proposed method. Data mean (% Area) was estimated for benzaldehyde (2.62%), 3,5,5-trimethylcclohex-2-en-1-one (6.14%), 2,6,6-trimethylcyclohex-2-ene-1,4-dione (1.90%), 2-hydroxy-3,5,5-trimethylcyclohex-2-en-1-one (1.29%), 2,6,6-trimethylcyclohexa-1,3-diene-1-carbaldehyde (1.75%), 4-methoxybenzaldehyde (3.62%), 3,4,5-trimethylphenol (0.98%), 1,1,5-trimethyl-1,2-dihydronaphthalene (1.21%), (E)-1-(2,6,6-trimethylcyclohexa-1,3-dien-1-yl)but-2-en-1-one (0.84%), (E)-1,6,6-trimethyl-7-(3-oxobut-1-en-1-yl)-3,8-dioxatricyclo[5.1.0.02,4]octan-5-one (0.60%), ethyl 4-methoxybenzoate (0.43%), (E)-4-(2,6,6-trimethylcyclohexa-1,3-dien-1-yl)but-3-en-2-one (0.37%), and 4,6,10,10-tetramethyl-5-oxatricyclo[4.4.0.01,4]dec-2-en-7-ol (0.37%).

**Figure 1:**
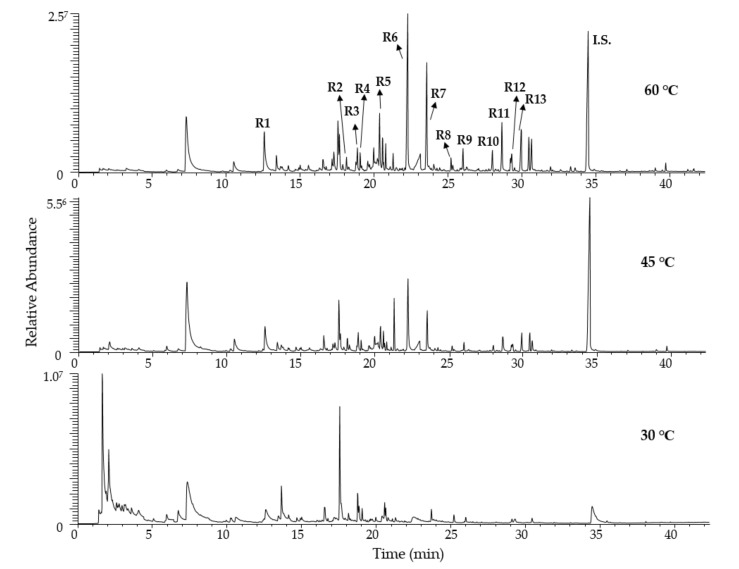
Chromatograms of the same sample at different temperatures (30, 45, and 60 °C).

The extraction temperature indicated a notable effect on total volatility (Figure 1). The ideal temperature for the isolation of compounds with lower molecular weight and high volatility was 30 °C. Contrariwise, 60 °C was better for molecules with lower volatility (Table 6). In our case, the temperature of 60 °C was selected. Considering the above results, the higher the extraction temperature the larger the partition coefficients of compounds [14]. Nonetheless, this relation was not linear because higher temperatures may lead to the formation of by-products or thermal decomposition [20]. 

**Figure 2:**
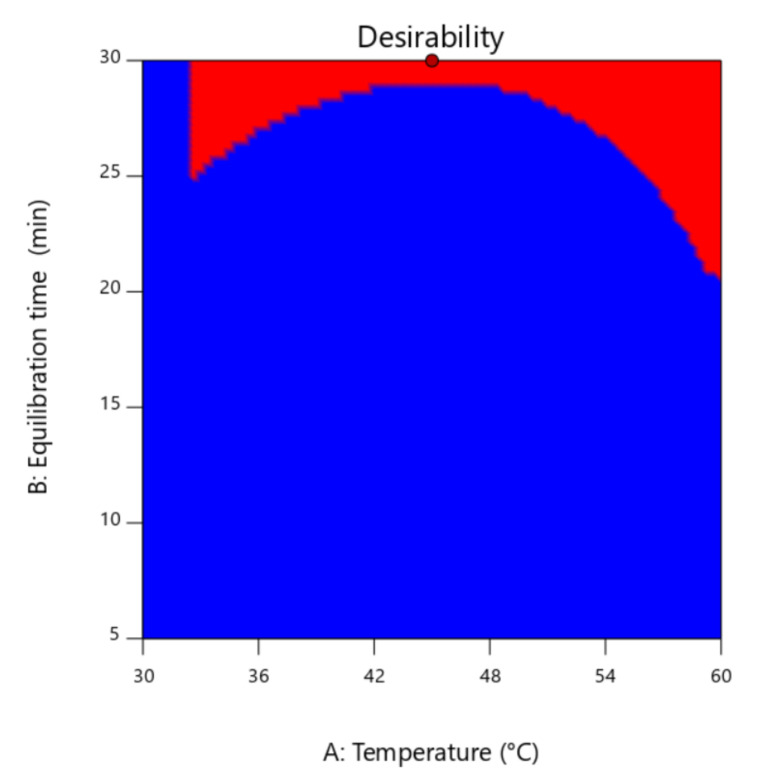
Desirability contour of equilibration time as a function of temperature of all responses.

The equilibration time, also known as “thermostating time”, showed an uncommon high value at 30 min. Figure 2 presents the desirability surface area of all responses. Due to the nature and molecular structure variability of autumn heather honey volatiles, compounds with short equilibration time can be displaced from the headspace of the vial gradually, by compounds with higher equilibration time. However, it is not a factor that significantly affects the efficiency of the system [14], since it reacts with other parameters at a relative moderate impact, as observed from the ANOVA test (Table 5).

Extraction time is critical for the sample to establish equilibrium with the SPME fiber coating. A typically 15 min extraction time showed a good performance. This parameter depends on the interactions of the molecules and can be lower when using the headspace technique and interact with high concentration samples. At the same time, it should be considered that many volatiles encumber the overall sensitivity and drive the specific compounds out of the fiber, which is easier to happen at prolonged extraction. On the other side, some studies indicated that the efficiency increases together with the extension of extraction time [14]. Obviously, the assessment is very difficult due to the intrinsic variability of honeys.

The magnetic stirrer speed, as previously referred to by Xagoraris [20], was confirmed to interact with most of the responses. However, this contribution is not always important as shown by the coded equation. Although one can assume that compounds with lower volatility require greater velocities, and in our case, 100 rpm gave satisfactory results. Thus, maintaining consistent agitation improves the accuracy and precision of the system.

The optimum sample volume was 6 mL, while this parameter had minimum impact on the isolation. Nevertheless, sensitivity is better when the headspace volume is small and fiber extracts faster compounds with higher volatility [37]. 

Finally, the ratio of water: Honey was proven a significant parameter. Honey is highly viscous and the addition of water enables the sample agitation, while also water evaporation drifts more easily the volatiles from honey. However, the excessive addition of water tends to dilute the honey reducing the concentration of specific molecules. The most favorable water: Honey ratio was 1:3 (*v*/*w*).

Reviewing the literature, in other botanical origin honey samples, different optimization conditions are reported. Ceballos [38] suggested the analytical conditions of the optimized SPME method by RSM as 60 μm PDMS/DVB fiber, 6 g honey, 3 mL water, and 20% *w*/*w* sodium chloride, 20 min for thermostatic time, 30 min for extraction at 60 °C. Plutowska [14] referenced the following conditions: CAR/PDMS/DVB fiber, 5:1 *w*/*w* honey to water ratio, 3 g sample, equilibration time 10 min, extraction time 30–60 min, and temperature at 60 °C. Bianchi [39] examined four different sets of conditions for volatile fraction isolation from thistle honey. Bianchin [40] proposed a new optimization strategy based on the use of three different extraction temperatures (60, 40, and 30 °C) followed by equilibration time (60, 36, and 6 min), respectively, in a single assay. Robotti, [41] reported as optimal extraction conditions for multi-floral honeys (extraction temperature: 70 °C; extraction time: 60 min; salt percentage: 27.50% *w*/*w*). Da Costa [42] reported the optimum condition for extraction of volatile compounds were as follows: equilibration time of 15 min, extraction time of 45 min, and extraction temperature of 45 °C. 

On the basis of the above considerations, it is difficult to predict the factors that can affect the isolation of each volatile compound of honey. In a previous study, the optimized combination of isolation conditions of thyme honey was different with temperature (60 °C), equilibration time (15 min), extraction time (30 min), magnetic velocity speed (700 rpm), sample volume (6 mL) and water honey ratio (1:3 *v*/*w*) [20]. Each honey has its unique composition thus requiring different optimization conditions regarding volatile compounds.

## 4. Conclusions

In terms of this research, the volatile fraction of 25 honey samples from indigenous monofloral autumn heather honey was investigated. The most important compounds indicating the botanical origin of this honey are benzaldehyde, 3,5,5-trimethylcclohex-2-en-1-one, 2,6,6-trimethylcyclohex-2-ene-1,4-dione, 2-hydroxy-3,5,5-trimethylcyclohex-2-en-1-one, 2,6,6-trimethylcyclohexa-1,3-diene-1-carbaldehyde, 4-methoxybenzaldehyde, 3,4,5-trimethylphenol, 1,1,5-trimethyl-1,2-dihydronaphthalene, (E)-1-(2,6,6-trimethylcyclohexa-1,3-dien-1-yl)but-2-en-1-one, (E)-1,6,6-trimethyl-7-(3-oxobut-1-en-1-yl)-3,8-dioxatricyclo[5.1.0.02,4]octan-5-one, ethyl 4-methoxybenzoate, (E)-4-(2,6,6-trimethylcyclohexa-1,3-dien-1-yl)but-3-en-2-one, and 4,6,10,10-tetramethyl-5-oxatricyclo[4.4.0.01,4]dec-2-en-7-ol. These compounds were identified in almost all samples in significant concentrations, with few exceptions. Obviously, the assessment of a quantitative reference value is very difficult to be set due to endogenous or exogenous factors. Some of the above volatiles were previously reported in heather honey. However, the autumn heather honey from *E. manipuliflora* has not been previously investigated. Τhe main volatile compounds were analyzed using a well-suited RSM methodology and predictive models were created to evaluate each volatile separately. Moreover, preconized optimum conditions (A: 60 °C B: 30 min C: 15 min D: 100 rpm E: 6 mL F: 1:3 (*v*/*w*)) were proposed for all dominant volatiles. In addition, a validation set amplified the results by responsiveness. This study reinforces the more reliable characterization of the volatile profile of autumn heather honey, aiming at the assessment of its botanical origin. In addition, it investigates the most common isolation factors, in terms of their ability to isolate their aroma fraction with relative abundance. 

## Figures and Tables

**Table 1:** Independent experimental factors and design layout runs.

Run	A: Temperature	B: Equilibration Time	C: Extraction Time	D: Magnetic Stirrer Velocity	E: Sample Volume	F: Water: Honey Ratio
Units	°C	min	min	rpm	mL	*v*/*w*
1	30.0	5.0	15.0	700.0	4.0	1:3
2	30.0	5.0	60.0	400.0	2.0	1:1
3	30.0	5.0	15.0	100.0	6.0	1:1
4	30.0	5.0	30.0	700.0	6.0	3:1
5	30.0	5.0	15.0	100.0	2.0	1:3
6	30.0	15.0	15.0	100.0	4.0	3:1
7	30.0	15.0	60.0	700.0	6.0	1:3
8	30.0	15.0	15.0	700.0	2.0	1:1
9	30.0	30.0	30.0	400.0	6.0	1:3
10	30.0	30.0	60.0	100.0	2.0	1:3
11	30.0	30.0	60.0	700.0	6.0	1:1
12	30.0	30.0	60.0	100.0	6.0	3:1
13	30.0	30.0	15.0	700.0	6.0	3:1
14	30.0	30.0	30.0	100.0	4.0	1:1
15	30.0	30.0	60.0	700.0	2.0	3:1
16	45.0	5.0	60.0	100.0	6.0	1:3
17	45.0	5.0	60.0	700.0	2.0	1:3
18	45.0	5.0	15.0	400.0	4.0	1:1
19	45.0	15.0	60.0	100.0	2.0	1:1
20	45.0	15.0	60.0	700.0	6.0	3:1
21	45.0	30.0	15.0	100.0	6.0	1:3
22	45.0	30.0	30.0	100.0	2.0	3:1
23	45.0	30.0	15.0	700.0	2.0	1:3
24	60.0	5.0	60.0	100.0	2.0	3:1
25	60.0	5.0	15.0	100.0	6.0	3:1
26	60.0	5.0	60.0	700.0	6.0	1:1
27	60.0	5.0	30.0	100.0	2.0	1:1
28	60.0	5.0	15.0	700.0	6.0	1:3
29	60.0	5.0	60.0	400.0	6.0	3:1
30	60.0	5.0	15.0	700.0	2.0	3:1
31	60.0	15.0	30.0	400.0	2.0	1:3
32	60.0	30.0	60.0	100.0	6.0	1:1
33	60.0	30.0	30.0	700.0	4.0	3:1
34	60.0	30.0	15.0	100.0	2.0	1:1
35	60.0	30.0	15.0	700.0	6.0	1:1
36	60.0	30.0	60.0	700.0	6.0	1:3
37	60.0	30.0	60.0	700.0	2.0	1:1
38	60.0	30.0	15.0	100.0	4.0	1:3

**Table 2:** Volatile compounds isolated from headspace of autumn heather honey.

No.	Volatile Compounds	CAS Number	RT ^a^	RI ^b^	Min (mg kg^−1^)	Max (mg kg^−1^)	Average (mg kg^−1^)
Esters							
1	methyl benzoate	93-58-3	17.3	1093	0.00	0.33	0.02
2	methyl octanoate	111-11-5	18.3	1124	0.00	0.17	0.06
3	ethyl benzoate	93-89-0	19.6	1165	0.00	1.68	0.11
4	methyl 2-phenylacetate	101-41-7	19.8	1179	0.00	0.32	0.04
5	methyl 2-hydroxybenzoate (methyl salicylate)	119-36-8	20.4	1192	0.00	0.54	0.06
6	methyl nonanoate	1731-84-6	21.3	1222	0.06	0.44	0.16
7	methyl decanoate	110-42-9	24.3	1322	0.00	0.10	0.05
8	ethyl 4-methoxybenzoate (Ethyl anisate)	94-30-4	28.7	1458	0.00	0.24	0.02
9	methyl dodecanoate	111-82-0	30.8	1521	0.00	0.06	0.01
10	bis(2-methylpropyl) benzene-1,2-dicarboxylate	84-69-5	39.0	1859	0.00	0.07	0.02
Hydrocarbons							
11	octane	111-65-9	6.3	800	0.00	0.18	0.06
12	nonane	111-84-2	10.3	898	0.00	0.16	0.03
13	undecane	1120-21-4	17.6	1101	0.10	0.52	0.21
14	dodecane	112-40-3	20.7	1201	0.00	0.18	0.02
Alcohols							
15	oct-1-en-3-ol	3391-86-4	13.4	981	0.00	0.26	0.02
16	2-ethylhexan-1-ol	104-76-7	15.1	1029	0.00	0.16	0.03
17	5-(3,3-dimethyloxiran-2-yl)-3-methylpent-1-en-3-ol (*cis*-linalool oxide)	5989-33-3	16.6	1072	0.00	0.35	0.07
18	2-phenylethan-1-ol	60-12-8	17.9	1114	0.00	0.34	0.06
19	4-methyl-1-(prop-1-en-2-yl)cyclohex-3-en-1-ol (1,8-methadien-4-ol)	3419-02-1	20.0	1183	0.00	0.54	0.02
20	3,4,5-trimethylphenol	527-54-8	24.0	1314	0.00	0.93	0.08
21	4,6,10,10-tetramethyl-5-oxatricyclo[4.4.0.01,4]dec-2-en-7-ol	97371-50-1	29.3	1476	0.00	0.11	0.01
22	6,6-dimethyl-5-methylenebicyclo[2.2.1]heptan-2-ol (6-camphenol)	3570-04-5	30.5	1510	0.00	0.38	0.02
Aldehydes							
23	furan-2-carbaldehyde (furfural)	98-01-1	7.4	826	0.01	2.61	1.14
24	benzaldehyde	100-52-7	12.6	959	0.02	1.44	0.18
25	octanal	124-13-0	14.2	1001	0.00	0.15	0.05
26	2-phenylacetaldehyde	122-78-1	15.6	1041	0.00	0.85	0.16
27	nonanal	124-19-6	17.7	1104	0.07	0.46	0.19
28	2,6,6-trimethylcyclohexa-1,3-diene-1-carbaldehyde (safranal)	116-26-7	20.6	1198	0.00	0.54	0.12
29	decanal	112-31-2	20.8	1205	0.00	0.35	0.15
30	4-methoxybenzaldehyde (p-anisaldehyde)	123-11-5	22.4	1261	0.00	1.36	0.23
Ketones							
31	1-(furan-2-yl)ethan-1-one	1192-62-7	10.6	907	0.07	0.34	0.16
32	cyclohex-2-en-1-one	930-68-7	14.7	1015	0.00	0.15	0.01
33	3,5,5-trimethylcyclohex-2-en-1-one (a-isophorone)	78-59-1	18.2	1120	0.01	4.16	0.43
34	2,6,6-trimethylcyclohex-2-ene-1,4-dione (4-oxoisophorone)	1125-21-9	18.9	1143	0.01	0.89	0.13
35	2-hydroxy-3,5,5-trimethylcyclohex-2-en-1-one (2-hydroxyisophorone)	4883-60-7	19.0	1145	0.00	0.29	0.09
36	1-(1,4-dimethylcyclohex-3-en-1-yl)ethanone	43219-68-7	19.1	1149	0.00	0.22	0.02
37	(E)-1-(2,6,6-trimethylcyclohexa-1,3-dien-1-yl)but-2-en-1-one (β-damascenone)	23726-93-4	26.0	1377	0.00	0.16	0.05
38	(E)-4-(2,4,4-trimethylcyclohexa-1,5-dien-1-yl)but-3-en-2-one	187519 ^c^	27.6	1420	0.00	0.10	0.01
39	(E)-1,6,6-trimethyl-7-(3-oxobut-1-en-1-yl)-3,8-dioxatricyclo[5.1.0.02,4]octan-5-one	192009 ^c^	28.0	1437	0.00	0.23	0.05
40	(E)-4-(2,6,6-trimethylcyclohexa-1,3-dien-1-yl)but-3-en-2-one (Dehydro-beta-ionone)	1203-08-3	29.2	1474	0.00	0.10	0.01
41	1-(4-(tert-butyl)-2,6-dimethylphenyl)ethan-1-one	2040-10-0	33.3	1584	0.00	0.14	0.04
42	(E)-3,5,5-trimethyl-4-(3-oxobut-1-en-1-yl)cyclohex-2-en-1-one	20194-68-7	35.0	1654	0.00	0.09	0.02
Acids							
43	nonanoic acid	112-05-0	23.2	1288	0.00	0.27	0.11
Terpenoids							
44	1-methyl-4-propan-2-ylbenzene (p-cymene)	99-87-6	14.9	1022	0.00	0.15	0.01
45	1-methyl-4-(prop-1-en-2-yl)benzene (p-cymenene)	1195-32-0	17.2	1090	0.00	0.18	0.02
46	1-methoxy-4-propylbenzene (4-propylanisole)	104-45-0	23.5	1299	0.00	0.83	0.04
Others							
47	(2S,8aR)-2,5,5,8a-tetramethyl-3,5,6,8a-tetrahydro-2H-chromene	41678-29-9	23.9	1306	0.00	0.09	0.01
48	1,1,5-trimethyl-1,2-dihydronaphthalene	357258 ^c^	25.2	1352	0.00	0.19	0.08
49	8-isopropyl-1-methyl-1,2,3,4-tetrahydronaphthalene	81603-43-2	31.5	1535	0.00	0.11	0.05

^a^ RT: Retention time (min); ^b^ RI: Experimental retention index; ^c^ NIST#.

**Table 3:** Dominant volatile compounds (responses R1-R13).

Response	Volatile Compound	Min (%Area)	Max (%Area)	Mean (%Area)	Std. Dev.
R1	benzaldehyde	1.59	6.70	4.52	1.22
R2	3,5,5-trimethylcyclohex-2-en-1-one	0.00	1.11	0.59	0.36
R3	2,6,6-trimethylcyclohex-2-ene-1,4-dione	0.00	2.90	1.32	0.75
R4	2-hydroxy-3,5,5-trimethylcyclohex-2-en-1-one	0.53	2.88	1.42	0.55
R5	2,6,6-trimethylcyclohexa-1,3-diene-1-carbaldehyde	0.98	4.57	2.37	0.81
R6	4-methoxybenzaldehyde	0.00	13.52	6.25	4.07
R7	3,4,5-trimethylphenol	0.00	0.72	0.22	0.29
R8	1,1,5-trimethyl-1,2-dihydronaphthalene	0.00	6.09	2.00	1.53
R9	(E)-1-(2,6,6-trimethylcyclohexa-1,3-dien-1-yl)but-2-en-1-one	0.00	1.80	1.13	0.40
R10	(E)-1,6,6-trimethyl-7-(3-oxobut-1-en-1-yl)-3,8-dioxatricyclo[5.1.0.02,4]octan-5-one	0.00	1.38	0.63	0.53
R11	ethyl 4-methoxybenzoate	0.00	3.96	1.39	1.30
R12	(E)-4-(2,6,6-trimethylcyclohexa-1,3-dien-1-yl)but-3-en-2-one	0.00	2.18	0.37	0.48
R13	4,6,10,10-tetramethyl-5-oxatricyclo[4.4.0.01,4]dec-2-en-7-ol	0.00	1.35	0.75	0.40

**Table 4:** ANOVA, Box-Cox and determination of coefficient (R^2^) of each response subjected to the model.

	ANOVA (*p*-Value < 0.05)						Box-Cox			R^2^
Response	A *	B *	C *	D *	E *	F *	CI Low ^a^	Current Lambda	CI High ^a^	
R1	0.19	0.50	0.40	0.35	0.60	0.00	0.26	1.00	3.02	0.988
R2	0.23	0.84	0.11	0.05	0.04	0.01	−0.04	1.00	1.09	0.979
R3	0.09	0.12	0.54	0.19	0.44	0.00	0.28	1.00	1.24	0.988
R4	0.00	0.30	0.33	0.44	0.11	0.04	−1.63	1.00	2.28	0.932
R5	0.09	0.53	0.73	0.36	0.17	0.07	−2.22	1.00	2.35	0.895
R6	0.00	0.53	0.00	0.64	0.74	0.00	0.33	1.00	2.35	0.997
R7	0.39	0.05	0.74	0.52	0.35	0.00	−0.79	1.00	1.23	0.978
R8	0.00	0.41	0.37	0.07	0.59	0.00	0.00	1.00	1.88	0.985
R9	0.66	0.95	0.35	0.18	0.12	0.00	0.70	1.00	2.85	0.956
R10	0.00	0.39	0.97	0.24	0.32	0.09	−0.17	1.00	1.78	0.991
R11	0.00	0.32	0.01	0.70	0.23	0.00	−0.11	1.00	0.83	0.988
R12	0.11	0.31	0.66	0.18	0.98	0.72	−0.61	1.00	1.40	0.913
R13	0.29	0.04	0.08	0.13	0.37	0.01	0.30	1.00	2.02	0.986

^a^ 95% confidence interval level. * A: Temperature; B: Equilibration time; C: Extraction time; D: Magnetic stirrer velocity; E: Sample volume; F: water: honey ratio.

**Table 5:** Contingent combinations of the SPME conditions and final equation in terms of coded factors.

**R1**	MT ^a^	F	AD	DF	B^2^	D^2^			
	CE ^b^	+0.69	+0.31	+0.39	+0.75	−0.81			
**R2**	MT	E	F	AC	AF				
	CE	+0.08	+0.21	−0.13	−0.28				
**R3**	MT	F	AC	AF	CF	DE	A^2^	D^2^	
	CE	+0.56	−0.20	+0.14	+0.20	+0.13	−0.42	−0.43	
**R4**	MT	A	F						
	CE	−0.47	+0.21						
**R5**	MT	No significant model terms							
	CE								
**R6**	MT	A	C	F					
	CE	+0.99	+0.53	−0.14					
**R7**	MT	F	AB	AC	AD	BD	CF	A^2^	E^2^
	CE	−0.18	+0.10	+0.09	+0.08	−0.09	−0.06	+0.17	+0.31
**R8**	MT	A	F	AF					
	CE	−0.88	−0.12	+0.60					
**R9**	MT	F	A^2^						
	CE	+0.21	−0.39						
**R10**	MT	A	A^2^						
	CE	+0.50	−0.42						
**R11**	MT	A	C	F	AF				
	CE	+0.96	+0.27	+0.60	+0.40				
**R12**	MT	No significant model terms							
	CE								
**R13**	MT	B	F	AB	AF	BE	DE	E^2^	
	CE	−0.09	+0.16	−0.12	+0.13	+0.12	−0.11	−0.21	

^a^ MT: model term; ^b^ CE: coded equation.

**Table 6:** Optimum conditions, desirabilities and predicted mean for each dominant volatile compound.

Response	A *	B *	C *	D *	E *	F *	Desirability	Predicted Mean (%Area)
R1	60	5	60	700	6	1:1	1.000	4.07 ± 0.36
R2	45	30	15	700	2	1:3	1.000	0.96 ± 0.18
R3	60	30	15	100	4	1:3	1.000	1.63 ± 0.22
R4	60	30	15	100	4	1:3	1.000	0.85 ± 0.19
R5	45	30	15	100	6	1:3	1.000	1.97 ± 0.71
R6	60	15	15	100	2	1:3	1.000	12.61 ± 0.64
R7	45	30	15	100	6	1:3	1.000	0.11 ± 0.02
R8	60	5	15	700	6	1:3	1.000	0.64 ± 0.11
R9	60	15	30	400	2	1:3	1.000	0,77 ± 0.23
R10	60	30	30	100	4	1:3	1.000	0.84 ± 0.22
R11	60	30	15	100	2	1:1	1.000	2.48 ± 0.39
R12	60	30	60	700	6	1:3	1.000	1.09 ± 0.39
R13	60	30	15	700	6	1:1	1.000	0.98 ± 0.27

* A: Temperature; B: Equilibration time; C: Extraction time; D: Magnetic stirrer velocity; E: Sample volume; F: water: honey ratio.

## Data Availability

Not applicable.
